# *N*-Benzoyl-*N*′-phenyl­urea

**DOI:** 10.1107/S1600536810001807

**Published:** 2010-01-20

**Authors:** Andrzej Okuniewski, Jaroslaw Chojnacki, Barbara Becker

**Affiliations:** a Department of Inorganic Chemistry, Gdansk University of Technology, Narutowicza 11/12, 80-233 Gdańsk, Poland

## Abstract

In the title compound, C_14_H_12_N_2_O_2_, the mol­ecular conformation is determined by a strong intra­molecular N—H⋯O=C hydrogen bond. In the crystal, pairs of mol­ecules are connected by inter­molecular N—H⋯O=C hydrogen bonds, forming centrosymmetric dimers. No specific inter­actions between dimers could be found.

## Related literature

For related structures, see: Bart *et al.* (1989 ▶); Zhong *et al.* (1998 ▶); Moon *et al.* (2002 ▶), Yamin & Mardi (2003 ▶); Chen *et al.* (2004 ▶); Su (2005 ▶); Yan *et al.* (2007 ▶, 2008 ▶); Liu *et al.* (2008 ▶, 2008*a* ▶,*b* ▶). For graph-set notation, see: Etter (1990 ▶). The title compound was obtained as a byproduct during the synthesis of a copper(I) complex with *N*-benzoyl-*N′*-phenyl­thio­urea prepared according to Frank & Smith (1948 ▶).
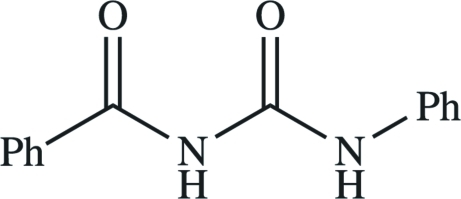


## Experimental

### 

#### Crystal data

C_14_H_12_N_2_O_2_
*M* *_r_* = 240.26
Monoclinic, 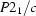

*a* = 15.5641 (8) Å
*b* = 4.6564 (3) Å
*c* = 21.1029 (15) Å
β = 128.716 (4)°
*V* = 1193.31 (15) Å^3^
*Z* = 4
Mo *K*α radiation
μ = 0.09 mm^−1^
*T* = 150 K
0.54 × 0.10 × 0.09 mm

#### Data collection

Oxford Diffraction Xcalibur diffractometer with a Sapphire2 (large Be window) detector
Absorption correction: analytical [*CrysAlis PRO* (Oxford Diffraction, 2009 ▶); analytical numeric absorption correction using a multi-faceted crystal model based on expressions derived by Clark & Reid (1995 ▶)] *T*_min_ = 0.971, *T*_max_ = 0.993
4425 measured reflections
2221 independent reflections
1575 reflections with *I* > 2σ(*I*)
*R*_int_ = 0.022

#### Refinement

*R*[*F* ^2^ > 2σ(*F* ^2^)] = 0.039
*wR*(*F* ^2^) = 0.098
*S* = 0.94
2221 reflections
211 parameters
Only H-atom coordinates refined
Δρ_max_ = 0.22 e Å^−3^
Δρ_min_ = −0.14 e Å^−3^

### 

Data collection: *CrysAlis PRO* (Oxford Diffraction, 2009 ▶); cell refinement: *CrysAlis PRO*; data reduction: *CrysAlis PRO* program(s) used to solve structure: *SHELXS97* (Sheldrick, 2008 ▶); program(s) used to refine structure: *SHELXL97* (Sheldrick, 2008 ▶); molecular graphics: *ORTEP-3 for Windows* (Farrugia, 1997 ▶); software used to prepare material for publication: *WinGX* (Farrugia, 1999 ▶) and *PLATON* (Spek, 2009 ▶).

## Supplementary Material

Crystal structure: contains datablocks global, I. DOI: 10.1107/S1600536810001807/im2175sup1.cif

Structure factors: contains datablocks I. DOI: 10.1107/S1600536810001807/im2175Isup2.hkl

Additional supplementary materials:  crystallographic information; 3D view; checkCIF report

## Figures and Tables

**Table 1:** Hydrogen-bond geometry (Å, °)

*D*—H⋯*A*	*D*—H	H⋯*A*	*D*⋯*A*	*D*—H⋯*A*
N1—H1⋯O2	0.93 (2)	1.85 (2)	2.634 (2)	140 (1)
N2—H2⋯O1^i^	0.93 (2)	1.97 (2)	2.882 (1)	169 (1)

Symmetry code: (i) 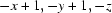.

**Fig. 1.**
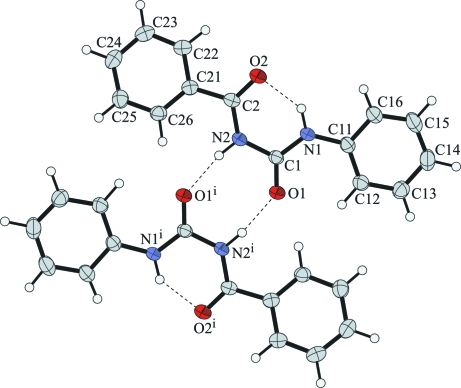
Structure of centrosymmetric N-benzoyl-N'-phenylurea dimer. Ellipsoids are drawn at 50% probability level. Symmetry code: (i) –x+1, –y+1, –z.

## supplementary crystallographic information

### Comment

*N*-Benzoyl-*N'*-phenylurea halogeno derivatives are used as pesticides. For instance 1-(3,5-dichloro-2,4-difluorophenyl)-3-(2,6-difluorobenzoyl)urea was found to act as chitin synthesis inhibitor (Zhong *et al.*,1998).

*N*-benzoyl-*N'*-phenylurea molecules adopt a conformation that allows formation of N—H···O═C intramolecular hydrogen bonds, denoted as R(6) in graph set notation (Etter, 1990). This conformation is commonly noted among all *N'*-monosubstituted or *N'*-unsubstituted *N*-benzoylureas and *N*-benzoylthioureas (see: related structures). Additional N—H···O═C intermolecular R^2^_2_(8) hydrogen bonds bind two urea derivative molecules to form a centrosymmetric dimer (Fig. 1), which is also common. Only one known structure does not exhibit such a motif (Moon *et al.*, 2002).

No π-π stacking interactions can be found in this structure (closest ring centroids distance is about 5.60 Å with the dihedral angle between the rings α about 80°).

The dihedral angle N1—C1—N2—C2 describing the twist of two amide subunits of urea derivatives is equal to 3.0 (2)°. This value is in the range known from other studies: from 0.07° (Yan *et al.*, 2007) to 7.45° (Su, 2005).

### Experimental

*N*-benzoyl-*N'*-phenylurea was obtained as a byproduct during the synthesis of a copper(I) complex with *N*-Benzoyl-*N'*-phenylthiourea prepared according to Frank & Smith (1948). Obviously the ligand underwent basic hydrolysis with CH_3_ONa in acetone. Colourless single crystals suitable for X-ray diffraction analysis were isolated from the reaction mixture after it was kept at room temperature for a few days. From 5.12 g (0.02 mol) of thiourea derivative 0.87 g of *N*-benzoyl-*N'*-phenylurea was obtained. Yield: 18%.

### Refinement

All hydrogen atoms were found from difference Fourier map and refined without constraints.

### Crystal data

**Table:**

C_14_H_12_N_2_O_2_	*F*(000) = 504
*M**_r_* = 240.26	*D*_x_ = 1.337 Mg m^−^^3^
Monoclinic, *P*2_1_/*c*	Melting point: 482(2) K
Hall symbol: -P 2ybc	Mo *K*α radiation, λ = 0.71073 Å
*a* = 15.5641 (8) Å	Cell parameters from 2481 reflections
*b* = 4.6564 (3) Å	θ = 2.5–28.4°
*c* = 21.1029 (15) Å	µ = 0.09 mm^−^^1^
β = 128.716 (4)°	*T* = 150 K
*V* = 1193.31 (15) Å^3^	Needle, colourless
*Z* = 4	0.54 × 0.10 × 0.09 mm

### Data collection

**Table:**

Oxford Diffraction Xcalibur diffractometer with a Sapphire2 (large Be window) detector	2221 independent reflections
Radiation source: Mo Kα radiation	1575 reflections with *I* > 2σ(*I*)
graphite	*R*_int_ = 0.022
Detector resolution: 8.1883 pixels mm^-1^	θ_max_ = 25.5°, θ_min_ = 2.5°
ω scans	*h* = −18→9
Absorption correction: analytical [*CrysAlis PRO* (Oxford Diffraction, 2009); analytical numeric absorption correction using a multi-faceted crystal model based on expressions derived by Clark & Reid (1995)]	*k* = −3→5
*T*_min_ = 0.971, *T*_max_ = 0.993	*l* = −21→25
4425 measured reflections	

### Refinement

**Table:**

Refinement on *F*^2^	Primary atom site location: structure-invariant direct methods
Least-squares matrix: full	Secondary atom site location: difference Fourier map
*R*[*F*^2^ > 2σ(*F*^2^)] = 0.039	Hydrogen site location: difference Fourier map
*wR*(*F*^2^) = 0.098	Only H-atom coordinates refined
*S* = 0.94	*w* = 1/[σ^2^(*F*_o_^2^) + (0.0656*P*)^2^] where *P* = (*F*_o_^2^ + 2*F*_c_^2^)/3
2221 reflections	(Δ/σ)_max_ < 0.001
211 parameters	Δρ_max_ = 0.22 e Å^−^^3^
0 restraints	Δρ_min_ = −0.14 e Å^−^^3^

### Special details

**Table:**

Geometry. All s.u.'s (except the s.u. in the dihedral angle between two l.s. planes) are estimated using the full covariance matrix. The cell s.u.'s are taken into account individually in the estimation of s.u.'s in distances, angles and torsion angles; correlations between s.u.'s in cell parameters are only used when they are defined by crystal symmetry. An approximate (isotropic) treatment of cell s.u.'s is used for estimating s.u.'s involving l.s. planes.
Refinement. Refinement of *F*^2^ against ALL reflections. The weighted *R*-factor *wR* and goodness of fit *S* are based on *F*^2^, conventional *R*-factors *R* are based on *F*, with *F* set to zero for negative *F*^2^. The threshold expression of *F*^2^ > 2σ(*F*^2^) is used only for calculating *R*-factors(gt) *etc*. and is not relevant to the choice of reflections for refinement. *R*-factors based on *F*^2^ are statistically about twice as large as those based on *F*, and *R*- factors based on ALL data will be even larger.

### Fractional atomic coordinates and isotropic or equivalent isotropic displacement parameters (Å^2^)

**Table:**

	*x*	*y*	*z*	*U*_iso_*/*U*_eq_	
O1	0.58625 (7)	0.6582 (2)	−0.00745 (6)	0.0384 (3)	
O2	0.82393 (7)	0.2951 (2)	0.21526 (6)	0.0396 (3)	
N1	0.77093 (9)	0.6517 (3)	0.09887 (7)	0.0295 (3)	
N2	0.64890 (9)	0.3566 (3)	0.09771 (7)	0.0284 (3)	
C1	0.66640 (10)	0.5660 (3)	0.05886 (8)	0.0274 (3)	
C2	0.72519 (10)	0.2381 (3)	0.17319 (8)	0.0282 (3)	
C11	0.81196 (11)	0.8530 (3)	0.07373 (9)	0.0297 (4)	
C12	0.74750 (12)	0.9966 (3)	−0.00011 (9)	0.0330 (4)	
C13	0.79673 (13)	1.1897 (4)	−0.01876 (10)	0.0393 (4)	
C14	0.90876 (14)	1.2390 (4)	0.03479 (11)	0.0469 (5)	
C15	0.97228 (13)	1.0949 (4)	0.10784 (12)	0.0516 (5)	
C16	0.92476 (12)	0.9038 (4)	0.12780 (11)	0.0411 (4)	
C21	0.68355 (10)	0.0351 (3)	0.20329 (8)	0.0275 (3)	
C22	0.75435 (12)	−0.0253 (3)	0.28626 (9)	0.0336 (4)	
C23	0.72257 (13)	−0.2128 (4)	0.31894 (10)	0.0389 (4)	
C24	0.61969 (13)	−0.3423 (4)	0.26921 (10)	0.0390 (4)	
C25	0.54898 (12)	−0.2851 (3)	0.18661 (10)	0.0368 (4)	
C26	0.58046 (11)	−0.0960 (3)	0.15362 (9)	0.0316 (4)	
H1	0.8205 (12)	0.559 (3)	0.1480 (10)	0.039 (4)*	
H13	0.7502 (12)	1.291 (4)	−0.0711 (10)	0.041 (4)*	
H12	0.6663 (12)	0.961 (3)	−0.0395 (8)	0.032 (4)*	
H2	0.5745 (13)	0.328 (3)	0.0717 (9)	0.041 (4)*	
H23	0.7751 (12)	−0.257 (3)	0.3782 (10)	0.040 (4)*	
H26	0.5289 (12)	−0.059 (3)	0.0951 (9)	0.032 (4)*	
H22	0.8256 (13)	0.077 (4)	0.3210 (9)	0.044 (4)*	
H25	0.4762 (13)	−0.375 (3)	0.1504 (9)	0.043 (4)*	
H24	0.5963 (12)	−0.477 (4)	0.2916 (9)	0.043 (4)*	
H14	0.9397 (13)	1.385 (4)	0.0187 (10)	0.056 (5)*	
H15	1.0520 (15)	1.117 (4)	0.1465 (10)	0.060 (5)*	
H16	0.9654 (14)	0.800 (4)	0.1778 (11)	0.055 (5)*	

### Atomic displacement parameters (Å^2^)

**Table:**

	*U*^11^	*U*^22^	*U*^33^	*U*^12^	*U*^13^	*U*^23^
O1	0.0223 (5)	0.0518 (7)	0.0288 (6)	−0.0014 (4)	0.0100 (5)	0.0106 (5)
O2	0.0231 (5)	0.0527 (7)	0.0304 (6)	−0.0018 (5)	0.0107 (5)	0.0068 (5)
N1	0.0205 (6)	0.0363 (7)	0.0235 (6)	−0.0025 (5)	0.0097 (5)	0.0026 (6)
N2	0.0199 (6)	0.0351 (7)	0.0239 (6)	−0.0017 (5)	0.0106 (5)	0.0007 (6)
C1	0.0234 (7)	0.0333 (8)	0.0226 (7)	−0.0020 (6)	0.0130 (6)	−0.0018 (7)
C2	0.0237 (7)	0.0320 (8)	0.0243 (7)	0.0018 (6)	0.0128 (6)	−0.0015 (7)
C11	0.0275 (7)	0.0316 (8)	0.0297 (8)	−0.0041 (6)	0.0178 (6)	−0.0048 (7)
C12	0.0308 (8)	0.0373 (9)	0.0289 (8)	−0.0040 (6)	0.0178 (7)	−0.0030 (7)
C13	0.0443 (9)	0.0417 (10)	0.0360 (9)	−0.0046 (7)	0.0270 (8)	−0.0006 (8)
C14	0.0473 (10)	0.0458 (11)	0.0562 (11)	−0.0118 (8)	0.0366 (9)	−0.0009 (9)
C15	0.0303 (9)	0.0560 (12)	0.0579 (12)	−0.0089 (8)	0.0223 (9)	0.0049 (10)
C16	0.0273 (8)	0.0438 (10)	0.0413 (10)	−0.0041 (7)	0.0162 (7)	0.0051 (9)
C21	0.0279 (7)	0.0267 (8)	0.0276 (7)	0.0058 (6)	0.0173 (6)	0.0011 (7)
C22	0.0333 (8)	0.0339 (9)	0.0286 (8)	0.0037 (6)	0.0169 (7)	0.0011 (7)
C23	0.0441 (9)	0.0422 (10)	0.0296 (8)	0.0067 (7)	0.0227 (7)	0.0047 (8)
C24	0.0471 (9)	0.0367 (9)	0.0444 (9)	0.0052 (7)	0.0340 (8)	0.0080 (8)
C25	0.0357 (8)	0.0356 (9)	0.0425 (9)	0.0002 (7)	0.0261 (8)	0.0015 (8)
C26	0.0282 (7)	0.0355 (9)	0.0286 (8)	0.0033 (6)	0.0165 (7)	0.0026 (7)

### Geometric parameters (Å, °)

**Table:**

O1—C1	1.2308 (16)	C14—H14	1.007 (19)
O2—C2	1.2304 (15)	C15—C16	1.381 (2)
N1—C1	1.3419 (16)	C15—H15	0.975 (18)
N1—C11	1.4114 (18)	C16—H16	0.956 (19)
N1—H1	0.926 (16)	C21—C26	1.394 (2)
N2—C2	1.3731 (18)	C21—C22	1.3950 (19)
N2—C1	1.4058 (18)	C22—C23	1.382 (2)
N2—H2	0.930 (15)	C22—H22	0.988 (16)
C2—C21	1.494 (2)	C23—C24	1.388 (2)
C11—C12	1.388 (2)	C23—H23	0.998 (16)
C11—C16	1.3907 (19)	C24—C25	1.386 (2)
C12—C13	1.388 (2)	C24—H24	0.983 (17)
C12—H12	1.001 (14)	C25—C26	1.388 (2)
C13—C14	1.380 (2)	C25—H25	0.980 (16)
C13—H13	0.984 (17)	C26—H26	0.979 (15)
C14—C15	1.378 (3)		
			
C1—N1—C11	128.07 (12)	C14—C15—C16	120.59 (15)
C1—N1—H1	113.5 (9)	C14—C15—H15	122.2 (10)
C11—N1—H1	118.4 (9)	C16—C15—H15	117.2 (11)
C2—N2—C1	127.79 (11)	C15—C16—C11	120.10 (16)
C2—N2—H2	118.7 (9)	C15—C16—H16	123.5 (10)
C1—N2—H2	112.3 (10)	C11—C16—H16	116.4 (10)
O1—C1—N1	125.41 (14)	C26—C21—C22	119.31 (14)
O1—C1—N2	118.47 (11)	C26—C21—C2	123.99 (13)
N1—C1—N2	116.12 (12)	C22—C21—C2	116.69 (13)
O2—C2—N2	122.27 (13)	C23—C22—C21	120.37 (14)
O2—C2—C21	120.53 (12)	C23—C22—H22	121.3 (9)
N2—C2—C21	117.20 (12)	C21—C22—H22	118.3 (9)
C12—C11—C16	119.63 (14)	C22—C23—C24	120.06 (15)
C12—C11—N1	124.25 (12)	C22—C23—H23	119.3 (9)
C16—C11—N1	116.12 (13)	C24—C23—H23	120.7 (9)
C11—C12—C13	119.38 (14)	C25—C24—C23	120.03 (15)
C11—C12—H12	120.2 (8)	C25—C24—H24	119.1 (9)
C13—C12—H12	120.4 (8)	C23—C24—H24	120.9 (9)
C14—C13—C12	120.96 (16)	C24—C25—C26	120.07 (15)
C14—C13—H13	120.3 (9)	C24—C25—H25	121.3 (9)
C12—C13—H13	118.7 (9)	C26—C25—H25	118.6 (9)
C15—C14—C13	119.33 (16)	C25—C26—C21	120.16 (14)
C15—C14—H14	123.1 (10)	C25—C26—H26	118.5 (9)
C13—C14—H14	117.5 (10)	C21—C26—H26	121.4 (9)
			
N1—C1—N2—C2	3.0 (2)		

### Hydrogen-bond geometry (Å, °)

**Table:**

*D*—H···*A*	*D*—H	H···*A*	*D*···*A*	*D*—H···*A*
N1—H1···O2	0.93 (2)	1.85 (2)	2.634 (2)	140 (1)
N2—H2···O1^i^	0.93 (2)	1.97 (2)	2.882 (1)	169 (1)

Symmetry codes: (i) −*x*+1, −*y*+1, −*z*.
